# A Difficult Differential Diagnosis of Acute Cholecystitis in a Patient With Steroid-induced Diabetes

**DOI:** 10.4021/jocmr752w

**Published:** 2011-11-10

**Authors:** Yoshinori Masui, Akahito Sako, Naonori Tsuda, So Nishimura, Yasuji Seyama, Masato Nishida, Junichi Shindo, Takaaki Sakamoto, Hiroshi Kaneko, Hidekatsu Yanai

**Affiliations:** a Department of Internal Medicine, National Center for Global Health and Medicine, Kohnodai Hospital, Chiba 272-8516, Japan; b Clinical Research Center, National Center for Global Health and Medicine, Kohnodai Hospital, Chiba 272-8516, Japan; c Department of Surgery, Tokyo Metropolitan Bokuto Hospital, Tokyo 130-8575, Japan; d Department of Rheumatology, National Center for Global Health and Medicine, Kohnodai Hospital, Chiba 272-8516, Japan

## Abstract

An impairment of gallbladder motility due to autonomic neuropathy may cause cholestasis and result in gallbladder stone formation. Diabetes is one of risk factors for acute cholecystitis. Diabetes and steroid use are associated with the susceptibility to bacterial infections, we are apt to diagnose steroid-induced diabetic patients manifesting symptoms of cholecystitis as having acute bacterial infective cholecystitis. Here, we report a very rare steroid-induced diabetic patient complicated with gallbladder torsion-induced necrotizing cholecystitis due to a floating gallbladder.

**Keywords:**

Cholecystitis; Diabetes; Floating gallbladder; Torsion

## Introduction

Diabetes has been reported to be one of risk factors for acute cholecystitis and complicated clinical course in patients with symptomatic cholelithiasis [1]. Gallbladder motility is significantly impaired in diabetic patients due to autonomic neuropathy as compared with healthy subjects [2-4]. An impairment of gallbladder motility may cause cholestasis and result in gallbladder stone growth. Since diabetes and steroid use are associated with the susceptibility to infections, we are apt to diagnose steroid-induced diabetic patients manifesting symptoms of cholecystitis as having acute bacterial infective cholecystitis. Here, we show a very rare steroid-induced diabetic patient complicated with gallbladder torsion-induced necrotizing cholecystitis due to a floating gallbladder.

## Case Report

**Figure 1:**
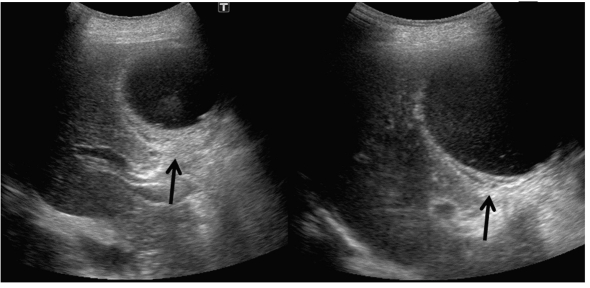
Abdominal ultrasound showed enlarged and distended gallbladder containing debris, and thickness of the wall of gallbladder.

**Figure 2:**
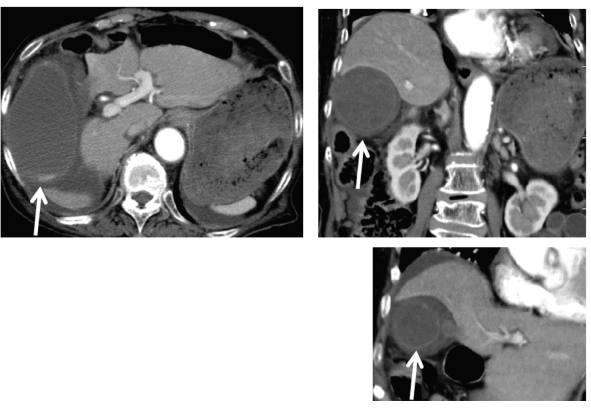
Abdominal enhanced computed tomography showed enlarged gallbladder and thickness of the gallbladder wall, and ascites around gallbladder.

**Figure 3:**
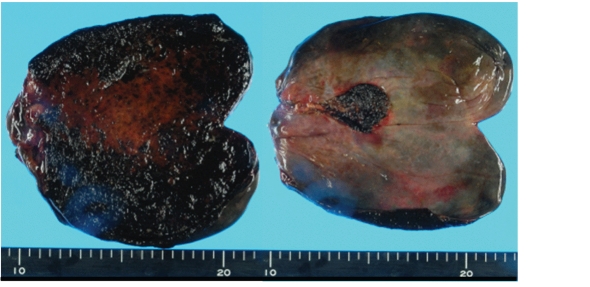
Abdominal operation showed the necrosis of gallbladder by torsion due to a floating gallbladder.

An 84-year-old woman was admitted to our hospital due to fever, abdominal pain and nausea, in May, 2011. At the age of 81 she has been diagnosed as Churg-Strauss syndrome and has been treated by prednisolone (7.5 - 10 mg/day). After the steroid treatment started, she developed steroid-induced diabetes, and her diabetes has been treated by premixed insulin (insulin lispro mix 50/50, 14 units before breakfast and 6 units before dinner). On the admission, her body temperature was 37.9 ^o^C and blood pressure was 154/95 mmHg. Physical examination revealed pain and muscular defense in her abdomen. Her body weight was 43 kg and height 148 cm (BMI 19.6 kg/m^2^). Fasting plasma glucose level (150 mg/dl) and hemoglobin A1C level (6.8%; normal range, 4.3 - 5.8%) were elevated. Laboratory data showed increased leukocyte counts (23,600/μl). Serum levels of asparatate aminotransferase (73 U/l; normal range, 10 - 40 U/l), alkaline phosphatase (374 U/L; normal range, 115 - 359 U/L), γ-glutamyl transpeptidase (61 U/L; normal range, < 30 U/L) and C-reactive protein (15.1 mg/dl; normal range, 0 - 0.3 mg/dl) were significantly elevated. Abdominal ultrasound showed enlarged and distended gallbladder containing debris, and thickness of the wall of gallbladder (Fig. 1). Abdominal enhanced computed tomography (CT) also revealed enlarged gallbladder and thickness of the gallbladder wall, and ascites around gallbladder, however, CT did not show a conical structure connecting the gallbladder to the liver (Fig. 2). From abdominal ultrasound and CT findings, leukocytosis and her history of diabetes and steroid use, we diagnosed her as having peritonitis due to acute bacterial cholecystitis or perforation of gallbladder, and she was referred to the department of Surgery. Abdominal operation revealed the necrosis of gallbladder by torsion due to a floating gallbladder (Fig. 3). The perforation of gallbladder wall was not detected.

## Discussion

Diabetic autonomic neuropathy causes gallbladder dysfunction [2]. Real-time sonography demonstrated that an impairment of gallbladder motility in type 1 and type 2 diabetic patients [3,4]. An impairment of gallbladder motility due to autonomic neuropathy may cause cholestasis and result in gallbladder stone formation and growth. A retrospective cohort study found an increased risk of biliary diseases in patients with type 2 diabetes [5]. Furthermore, diabetes has been reported to be one of risk factors for acute cholecystitis and a complicated clinical course in patients with symptomatic cholelithiasis [1]. Therefore, we are apt to diagnose diabetic patients manifesting symptoms of cholecystitis as having cholecystitis due to bacterial infection and cholelithiasis. In our case, the history of steroid use also leads us to diagnose her as having acute bacterial infective cholecystitis. The presence of leukocytosis and debris in abdominal ultrasound also supported the diagnosis of acute bacterial infective cholecystitis. However, an abdominal operation demonstrated that necrosis of gallbladder by torsion due to a floating gallbladder.

Torsion of the gallbladder is an extremely rare cause of acute surgical abdomen [6]. The gallbladder torsion is defined as the rotation of the gallbladder on its mesentery along the axis of the cystic duct and cystic artery [7]. The presence of a floating gallbladder, a redundant mesentery, is a prerequisite for the gallbladder torsion [7]. This disease manifests symptoms mimicking acute infective cholecystitis, therefore, preoperative diagnosis of this disease is difficult and the definitive diagnosis is usually made during surgery [7]. Actually, the definitive diagnosis of our case was also done by surgery. The presence of a conical structure connecting the gallbladder to the liver in enhanced abdominal CT has been reported to be a useful diagnostic clue for the gallbladder torsion, however, the conical structure was not detected in abdominal CT of our patient [8].

Although the etiology of gallbladder torsion remains unknown, the elderly and kyphoscoliosis which were observed in our patient, have been considered to be risk factors for the gallbladder torsion [8,9].

In conclusion, the gallbladder torsion is an emergent disease that must be immediately treated with cholecystectomy [7]. We should think of the development of the gallbladder torsion when we saw the elderly patients with kyphoscoliosis who manifesting symptoms of acute cholecystitis [8,9].
